# A *Caenorhabditis elegans* developmental decision requires insulin signaling-mediated neuron-intestine communication

**DOI:** 10.1242/dev.103846

**Published:** 2014-04

**Authors:** Wesley L. Hung, Ying Wang, Jyothsna Chitturi, Mei Zhen

**Affiliations:** 1 Lunenfeld-Tanenbaum Research Institute, University of Toronto, Toronto, Ontario, M5G 1X5, Canada; 2 Institute of Medical Science, University of Toronto, Toronto, Ontario, M5G 1X5, Canada; 3 Department of Molecular Genetics, University of Toronto, Toronto, Ontario, M5G 1X5, Canada

**Keywords:** EGL-3, KPC-1, Dauer formation, Insulin

## Abstract

Adverse environmental conditions trigger *C. elegans* larvae to activate an alternative developmental program, termed dauer diapause, which renders them stress resistant. High-level insulin signaling prevents constitutive dauer formation. However, it is not fully understood how animals assess conditions to choose the optimal developmental program. Here, we show that insulin-like peptide (ILP)-mediated neuron-intestine communication plays a role in this developmental decision. Consistent with, and extending, previous findings, we show that the simultaneous removal of INS-4, INS-6 and DAF-28 leads to fully penetrant constitutive dauer formation, whereas the removal of INS-1 and INS-18 significantly inhibits constitutive dauer formation. These ligands are processed by the proprotein convertases PC1/KPC-1 and/or PC2/EGL-3. The agonistic and antagonistic ligands are expressed by, and function in, neurons to prevent or promote dauer formation. By contrast, the insulin receptor DAF-2 and its effector, the FOXO transcription factor DAF-16, function solely in the intestine to regulate the decision to enter diapause. These results suggest that the nervous system normally establishes an agonistic ILP-dominant paradigm to inhibit intestinal DAF-16 activation and allow reproductive development. Under adverse conditions, a switch in the agonistic-antagonistic ILP balance activates intestinal DAF-16, which commits animals to diapause.

## INTRODUCTION

Organisms have developed various strategies to cope with adverse growth conditions. One common strategy to improve survival in challenging environments is to arrest metabolism and development (Adhikari et al., 2010; Podrabsky et al., 2010; Guidetti et al., 2011). Many insects enter diapause, an alternative developmental state, when facing unfavorable growth conditions (reviewed by Kostal, 2006). How animals assess their environmental conditions and choose developmental strategies accordingly is not fully understood.

Adverse conditions for individual or population growth trigger *C. elegans* larvae to activate an alternative developmental program, termed the dauer pathway or diapause (Golden and Riddle, 1982, 1984). Upon activation of the dauer pathway, *C. elegans* arrest reproductive development and remodel their metabolism and anatomy: they increase lipid storage, close off the mouth and sensilla, stop feeding and develop thick cuticles, which, cumulatively, promotes survival during prolonged dehydration and starvation (Cassada and Russell, 1975; Riddle et al., 1981; Vowels and Thomas, 1992). Since the first description of the dauer state (Cassada and Russell, 1975), several cellular and genetic mechanisms that govern dauer formation have been elucidated.

Cell ablation studies identified a small set of sensory neurons, ASI, ASJ and ADF, as crucial for activation or exit of the dauer state. Therefore, sensory processing governs the dauer decision (Albert et al., 1981; Bargmann and Horvitz, 1991). Genetic analyses of *C. elegans* mutants that either fail to activate dauer formation under adverse conditions (Dauer formation defective, or *Daf-d*) or form dauers constitutively regardless of environmental conditions (Dauer formation constitutive, or *Daf-c*), further revealed multiple signaling pathways that relay sensory information to influence the developmental decision (Riddle et al., 1981). Terminal execution of dauer formation is driven by the activation of a FOXO transcriptional factor, DAF-16 (Gottlieb and Ruvkun, 1994; Lin et al., 1997, 2001; Ogg et al., 1997; Paradis and Ruvkun, 1998; Paradis et al., 1999; Libina et al., 2003), and a nuclear hormone receptor (NHR), DAF-12 (Antebi et al., 1998, 2000). DAF-16 and DAF-12 activities are regulated by multiple signaling molecules, including insulin-like peptides (ILPs) (Pierce et al., 2001; Li et al., 2003; Cornils et al., 2011) and TGFβ (Ren et al., 1996; Schackwitz et al., 1996), from sensory neurons, as well as steroid dafachronic acids (DAs) primarily from neuroendocrine-like XXX cells (Motola et al., 2006; Schaedel et al., 2012). These signaling pathways therefore influence the choice between reproductive development and dauer formation (reviewed by Hu, 2007; Fielenbach and Antebi, 2008; Antebi, 2013; Ludewig and Schroeder, 2013). The interplay among these multiple signaling pathways, the mechanisms animals utilize to control DAF-12 and DAF-16 activity through these signaling pathways, and the transcriptional programs that underlie distinct developmental strategies remain to be fully elucidated.

*C. elegans* has a single ILP receptor (InR), DAF-2 (Kenyon et al., 1993; Kimura et al., 1997). DAF-2 activation initiates the phosphorylation of a kinase cascade, composed of PI3K/AGE-1 (Morris et al., 1996), PDK/PDK-1 and AKTs (AKT-1 and AKT-2) (Paradis and Ruvkun, 1998; Paradis et al., 1999), which leads to the phosphorylation and cytoplasmic retention of DAF-16 (Ogg et al., 1997; Henderson and Johnson, 2001; Lee et al., 2001a; Lin et al., 2001). DAF-2 and its downstream kinase cascade are essential for embryonic viability and also regulate postembryonic development, such as dauer formation, immunity, longevity, nervous system development and learning (reviewed by Kurz and Tan, 2004; Kaletsky and Murphy, 2010; Kenyon, 2010; Tissenbaum, 2012; Sasakura and Mori, 2013). Partial loss of function of DAF-2 and the kinase cascade promotes *Daf-c*. Removing the transcription factor DAF-16 prevents dauer formation in wild-type and insulin signaling-defective animals, even under adverse conditions (*Daf-d*). Therefore, insulin signaling prevents constitutive dauer formation through sequestering DAF-16.

The existence of 40 ILPs in *C. elegans* (Pierce et al., 2001; Li et al., 2003; Ritter et al., 2013) implies both functional specificity and redundancy. Consistent with functional redundancy, no single loss of an ILP gene leads to significant *Daf-c* (Ritter et al., 2013; this study), a phenotype exhibited by *daf-2* loss-of-function, temperature-sensitive (*lf;ts*) alleles (Gems et al., 1998). A *ts* gain-of-function (*gf*) mutation in one ILP, DAF-28, results in fully penetrant *Daf-c*, similar to severe *daf-2(lf;ts)* (Malone et al., 1996; Li et al., 2003; Cornils et al., 2011). *daf-28(gf)* was postulated to mimic the *daf-2 Daf-c* phenotype through non-specifically blocking ILP processing (Li et al., 2003). Overexpression of INS-4 or INS-6 partially supresses the *Daf-c* penetrance of *daf-28(gf)*, whereas overexpression of INS-1 or INS-18 exacerbates the *Daf-c* penetrance of weak *daf-2(lf;ts)* alleles (Pierce et al., 2001; Li et al., 2003; Cornils et al., 2011). INS-4/INS-6 and INS-1/INS-18 are therefore likely to be among the agonistic and antagonistic DAF-2 ligands that suppress and activate DAF-16, respectively, during dauer formation.

In this study, we address how insulin signaling regulates developmental decisions. We identified the cohort of ILPs and the processing enzymes that play essential roles in the insulin signaling-dependent dauer decision. We further examined where their effectors, DAF-2 and DAF-16, are required to repress or activate the dauer pathway. Results reveal that the nervous system secretes a specific cohort of ILPs to instruct the intestine to make the decision on dauer formation.

## RESULTS

### INS-4, INS-6 and DAF-28 function redundantly to inhibit dauer formation

**Fig. 1.**
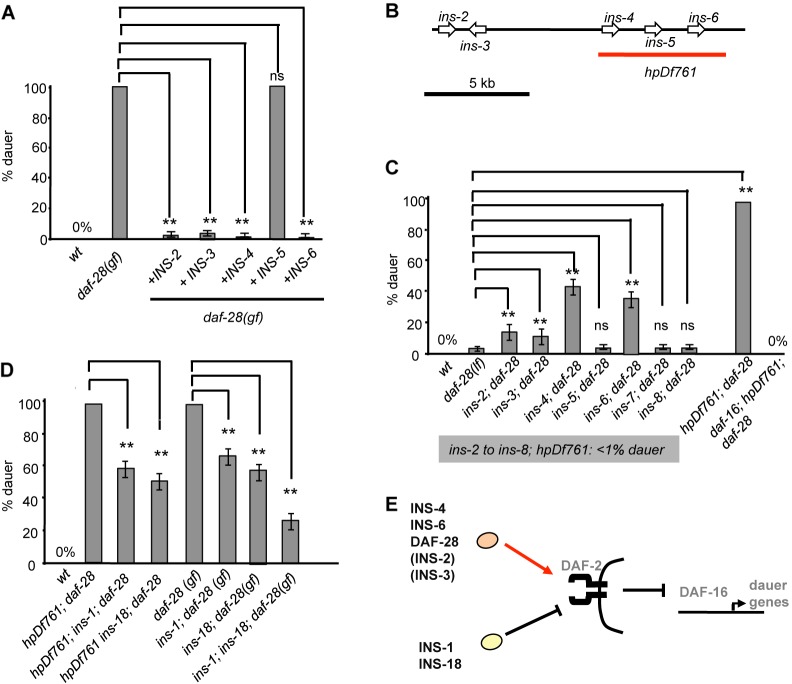
**INS-4, INS-6, DAF-28, INS-1 and INS-18 constitute the main dauer regulators.** (A) *daf-28(gf;ts)* exhibited 100% *Daf-c* (25°C). Overexpression of INS-2, INS-3, INS-4, INS-6, but not INS-5, suppressed the *Daf-c* penetrance of *daf-28(gf)* to varying degrees. (B) The genomic region from *ins-2* to *ins-6*. *hpDf761* harbors a 6 kb deletion of the indicated loci. (C) Agonistic ligands function redundantly to inhibit *Daf-c*. Deletion of *ins-2*, *ins-3*, *ins-4*, *ins-6* genes enhances *daf-28(lf) Daf-c*; *hpDf761;daf-28* exhibited 100% *Daf-c*. (D) Identification of antagonistic ligands. *ins-1* or *ins-18* deletion leads to partial suppression of *Daf-c* penetrance in *hpDf761;daf-28(lf)* and *daf-28(gf)*. (E) Summary of the main DAF-2 agonistic and antagonistic ligands that regulate dauer decisions. Proteins in parentheses denote minor agonists. **P<0.01 by the Tukey-Kramer comparison test. ns, not significant. Error bars, s.d. *N*>150 per strain, at least three repeats. Absence of error bar indicates that all trials had identical numbers.

Among the 35 available ILP deletion mutants, none exhibited significant *Daf-c* (Ritter et al., 2013; data not shown), implying functional redundancy among ILPs. We surveyed 40 ILP genes for genetic interactions with *daf-28(gf;ts)*. Consistent with previous findings on INS-4 and INS-6 (Li et al., 2003), we observed that overexpression of *ins-2*, *ins-3*, *ins-4* or *ins-6* from either pan-neural or endogenous promoters reduced the *Daf-c* penetrance of *daf-28(gf)* (Fig. 1A; supplementary material Fig. S1B). *ins-2*, *ins-3*, *ins-4* and *ins-6* deletion mutants also increased the *daf-28(lf) Daf-c* penetrance; *ins-4* and *ins-6* exhibited stronger enhancement (Fig. 1C).

These studies suggest that INS-4 and INS-6 are the main ILPs with functional redundancy, working together with DAF-28 to suppress dauer formation. As reported (Cornils et al., 2011), whereas neither *daf-28(lf)* nor *ins-6(lf)* exhibited detectable *Daf-c*, ∼30% of *ins-6(lf);daf-28(lf)* mutants were *Daf-c* (Fig. 1C). We also observed a similar degree of enhancement (∼40% *Daf-c*) in *ins-4(lf);daf-28(lf)* mutants (Fig. 1C). As *daf-2(lf;ts)* and *daf-28(gf;ts)* mutants exhibit fully penetrant *Daf-c*, we examined whether a complete loss of *daf-28*, *ins-4* and *ins-6(lf)* could recapitulate the dauer phenotype. We generated *hpDf761*, a 6 kb deletion across the *ins-4*, *ins-5* and *ins-6* loci (Fig. 1B). *hpDf761* did not exhibit detectable *Daf-c* (Table 1; supplementary material Fig. S2A). *hpDf761;daf-28(lf)* quadruple mutants, however, recapitulated the *Daf-c* penetrance of severe *daf-2(lf;ts)* alleles: they were 100% *Daf-c* at the non-permissive temperature (25°C); even at a permissive temperature for *daf-2(lf;ts)* (15°C), more than 80% of animals constitutively entered the dauer state (supplementary material Fig. S2A).

**Table 1.**
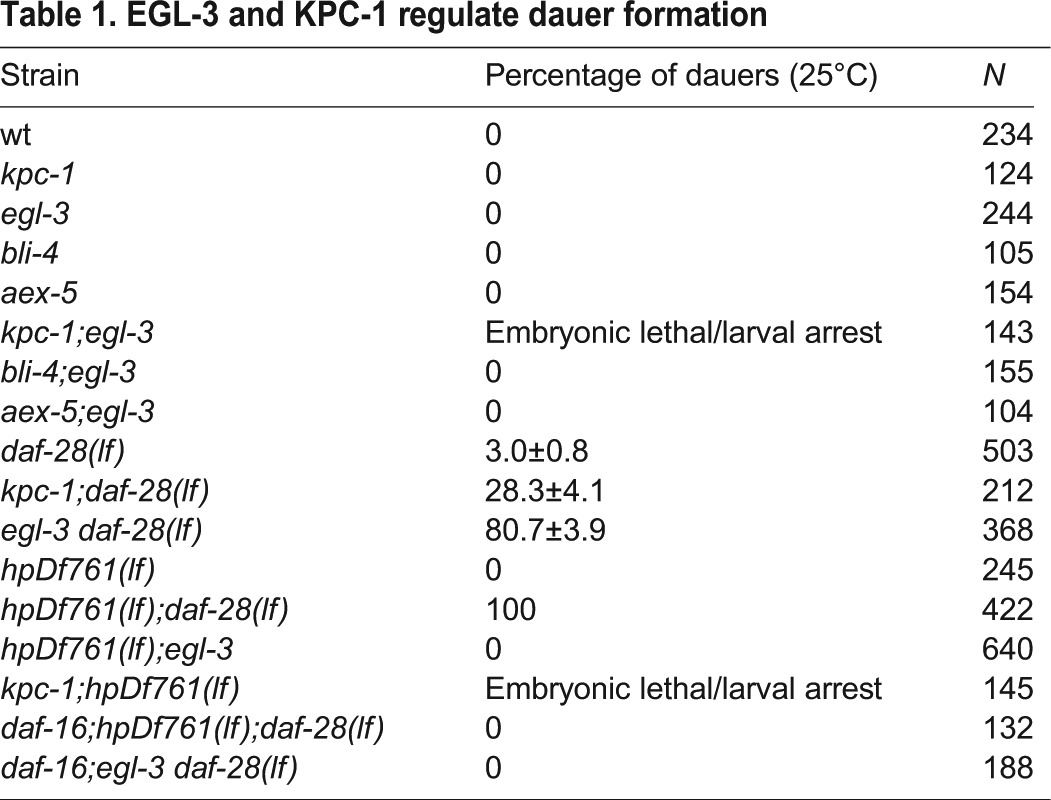
**EGL-3 and KPC-1 regulate dauer formation**

Although *hpDf761* deletes *ins-5* in addition to *ins-4* and *ins-6*, *ins-5* does not regulate dauer formation: *ins-5(lf)* did not enhance *Daf-c* of *daf-28(lf)* (Fig. 1C); overexpression of INS-5 did not suppress *Daf-c* of *daf-28(gf)* (Fig. 1A); restoring *ins-5* in *hpDf761;daf-28(lf)* did not rescue *Daf-c* (supplementary material Fig. S1A). Henceforth, we refer to *hpDf761* as a deletion mutant of *ins-4* and *ins-6* in the context of dauer formation.

The presence of ∼20% non-dauer *hpDf761;daf-28(lf)* population at 15°C (supplementary material Fig. S2A) indicates the existence of additional agonistic ILPs that inhibit dauer formation. Indeed, the constitutive overexpression of *ins-2* or *ins-3* suppressed *daf-28(gf) Daf-c* (Fig. 1A). *ins-2(lf);daf-28(lf)* and *ins-3(lf);daf-28(lf)* also exhibited a low but reproducible *Daf-c* population (∼15%) (Fig. 1C). We could not examine the effect of simultaneous removal of five agonistic ILPs, as we failed to delete the entire 12 kb region encoding the INS-2 to INS-6 cluster (Fig. 1B).

Like *daf-2(lf;ts)*, the *Daf-c* of *hpDf761;daf-28(lf)* was fully suppressed by *daf-16(lf;null)* (Fig. 1C). These results establish INS-4, INS-6 and DAF-28 as the main agonistic ILPs, and INS-2 and INS-3 the minor agonistic ILPs, that activate insulin signaling and prevent dauer formation (Fig. 1E).

### INS-1 and INS-18 are antagonistic ILPs in promoting dauer formation

Overexpression of either INS-1 or INS-18 causes *daf-2(lf;ts)* to exhibit *Daf-c* at permissive temperatures (Pierce et al., 2001). If INS-1 and INS-18 function as physiological, antagonistic ILPs, removing them should reduce the *Daf-c* penetrance in mutants with reduced insulin signaling.

Removing INS-1 or INS-18 in *hpDf761;daf-28(lf)* or *daf-28(gf;ts)* led to partial suppression of *Daf-c*, from ∼100% to ∼60%. Simultaneous removal of INS-1 and INS-18 in *daf-28(gf;ts)* further reduced its *Daf-c* to ∼30% (Fig. 1D), indicating an accumulative effect. These results confirm that INS-1 and INS-18 are physiological, antagonistic ligands that inhibit insulin signaling and promote dauer formation.

### KPC-1 and EGL-3 process agonistic ILPs

The 40 *C. elegans* ILPs are classified into three groups (Pierce et al., 2001; Li et al., 2003). The α group, comprising INS-1 and INS-18, adopts the conventional B-C-A conformation. The β group, comprising INS-2 to INS-9 and DAF-28, shares a non-conventional F-B-A configuration. Their maturation was predicted to involve C or F peptide processing. The remaining 30 ILPs adopt an integral B-A configuration that is unlikely to require any processing. Thus far, all identified agonistic ILPs that suppress dauers belong to the β group; the antagonistic ILPs that promote dauer formation belong to the α group. Whether they undergo processing is unknown.

There are four *C. elegans* proprotein convertases (PCs): three PC1 homologs, comprising AEX-5, BLI-4 and KPC-1 (Thacker and Rose, 2000; Thacker et al., 2000), and one PC2 homolog, EGL-3 (Kass et al., 2001). We developed an assay to monitor the *in vivo* processing of *C. elegans* ILPs and to identify the PC that mediates processing. Briefly, functional ILP reporters, generated by fusing GFP to the C-terminus of the A peptide, were expressed in wild type and PC mutants. If an ILP is processed, ILP::GFP should exhibit reduced mobility in the respective PC mutant as assessed by western blot analyses. The mobility shift, if representing an unprocessed ILP precursor, should be similar to that of a non-cleavable ILP reporter.

**Fig. 2.**
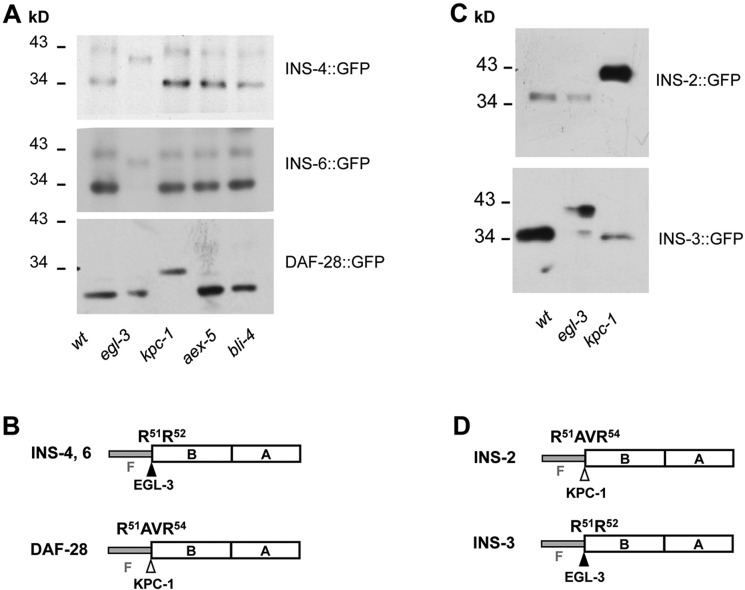
**EGL-3 and KPC-1 process different β subgroup agonistic ILPs.** (A) The migration pattern of INS-4::GFP, INS-6::GFP and DAF-28::GFP reporters in wild-type animals (wt), *egl-3*, *bli-4*, *aex-5* and *kpc-1* mutants. INS-4::GFP and INS-6::GFP showed reduced mobility in *egl-3*. The mobility of DAF-28::GFP was reduced in *kpc-1* mutants. (C) The mobility of INS-2::GFP and INS-3::GFP was reduced in *kpc-1* and *egl-3* mutants, respectively. (B,D) Illustration of the structure and processing of β group ILPs.

The β group includes all identified agonistic ILPs. Upon closer examination, we split them into two subgroups: INS-3, INS-4, INS-6, INS-7 and INS-9 harbor a consensus PC2-like cleavage sequence (RR or KR), whereas INS-2, INS-5 and DAF-28 have a PC1-like site (R-X-X-R) at the F-B junction. The F peptide of those ILPs with the PC2 site that were tested, namely INS-3, INS-4 and INS-6, was processed by EGL-3 (Fig. 2A,C) (Hung et al., 2013). These INS::GFP reporters exhibited reduced mobility in *egl-3* mutant lysate (Fig. 2A). The non-cleavable reporters for INS-4 and INS-6, in which the RR cleavage site was mutated to AA, exhibited the same mobility shift in wild-type lysate (Hung et al., 2013). By contrast, the F peptide of the subgroup with the PC1 site (INS-2 and DAF-28) was removed by KPC-1 (Fig. 2A,C). In both cases, EGL-3 and KPC-1 processing yielded an integral B-A peptide (Fig. 2B,D).

In brief, the β group agonistic ILPs INS-3, INS-4 and INS-6 are processed by EGL-3 whereas INS-2 and DAF-28 are processed by KPC-1.

### EGL-3 processes antagonistic INS-1

The α group ILPs, INS-1 and INS-18, adopt a proinsulin B-C-A configuration. In mammals, PC1 and PC2 cleave the B-C and C-A junctions, respectively, resulting in a B-A peptide linked by disulfide bonds (Bailyes et al., 1991; Malide et al., 1995).

**Fig. 3.**
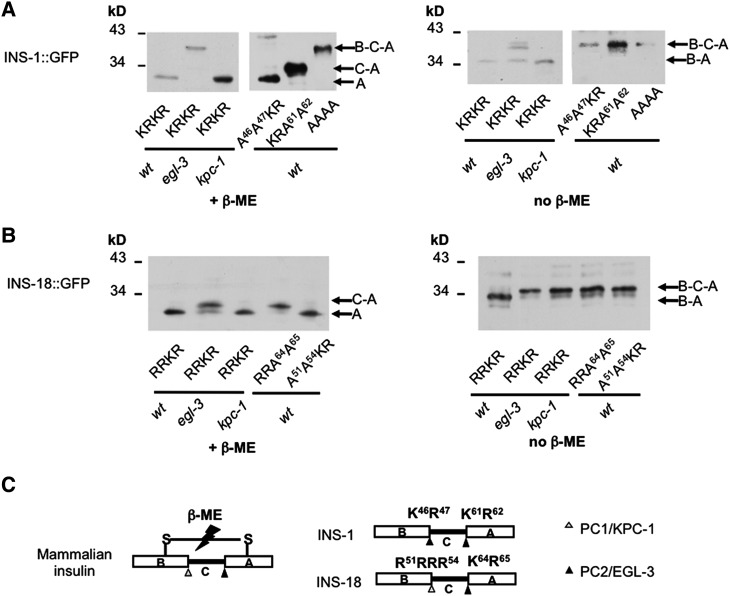
**EGL-3 and KPC-1 process α group antagonistic ILPs.** (A). The migration patterns of wild-type and mutated INS-1::GFP, expressed in wild-type animals (wt), and in *egl-3* and *kpc-1* mutants, under reducing (+β-ME) or non-reducing (−β-ME) conditions. Mutations of the cleavage sites (K46R47 and K61R62) prevent EGL-3-mediated processing at the respective junctions. (B) The migration pattern of wild-type and mutated INS-18::GFP in wild-type animals and in *egl-3* and *kpc-1* mutants under reducing or non-reducing conditions. Mutations at junctions, R51R54 and K64R65, block their processing by KPC-1 and EGL-3, respectively. (C) Comparison of the structure and processing of mammalian proinsulin, INS-1 and INS-18.

To determine whether the C peptide is processed in INS-1, we compared the migration of the INS-1::GFP reporter under reducing and non-reducing conditions (Fig. 3). INS-1::GFP, in the absence of β-mercaptoethanol (β-ME), should retain the B peptide (B-A::GFP). This results in a reduced migration compared with lysates treated with β-ME (A::GFP), as indeed observed in wild-type animals (Fig. 3A, KRKR in the wt lanes, left and right panels).

In *kpc-1* mutant background, the INS-1::GFP reporter exhibited identical migration patterns as in wild-type animals under both conditions (Fig. 3A, KRKR in *kpc-1* lanes). By contrast, in *egl-3* mutant background, the mobility difference under reducing and non-reducing conditions was abolished (Fig. 3A, KRKR in *egl-3* lanes, left and right panels). Importantly, INS-1::GFP in *egl-3* lysate exhibited reduced mobility compared with lysates from wild type and *kpc-1* mutants under both conditions (Fig. 3A, KRKR in *egl-3* lanes). These results suggest that the C peptide is removed in wild type and *kpc-1* but not in *egl-3* mutants. Hence, both B-C and C-A junctions may be processed by EGL-3 (Fig. 3A,C).

We identified PC2-like cleavage motifs, K46R47 and K61R62, at the B-C and C-A junctions of INS-1. We mutated them to non-cleavable forms and examined their mobility in lysates from wild-type animals (Fig. 3A). Under reducing conditions, INS-1(A46A47KR)::GFP, in which the B-C junction is non-cleavable but the C-A junction remains intact, exhibited the same mobility as the wild-type INS-1::GFP reporter (both producing A::GFP; Fig. 3A, left panel, A46A47KR in the wt lane). However, the INS-1(KRA61A62)::GFP reporter, in which only the C-A junction is non-cleavable, exhibited reduced migration compared with the wild-type INS-1::GFP (C-A::GFP versus A::GFP) (Fig. 3A, left panel, KRA61A62 in the wt lane). Under non-reducing conditions, mutant reporters for either or both junctions exhibited similar migration (B-C-A::GFP), which was slower than that of the wild-type reporter (B-A::GFP) (Fig. 3A, right panel, KRA61A62, A46A47KR and AAAA, wt lane). These results confirm the KR cleavage sites.

If EGL-3 were responsible for the cleavage, then the wild-type INS-1::GFP reporter in *egl-3* mutant background should exhibit an identical migration pattern as the non-cleavable INS-1(A46A47A61A62)::GFP reporter in lysates from wild-type animals, under both reducing and non-reducing conditions, and this is what we observed (Fig. 3, AAAA in *egl-3* lanes, both panels). In addition, in *egl-3* mutants, the wild-type reporter exhibited an identical migration pattern as the partial or non-cleavable reporters INS-1(A46A47KR), INS-1(KRA61A62) and INS-1(AAAA), under both reducing and non-reducing conditions (Fig. 3A). Therefore, EGL-3 processes INS-1 B-C (K46R47) and C-A (K61R62) junctions, resulting in a B-A peptide linked by disulfide bonds.

### EGL-3 and KPC-1 process antagonistic INS-18

Employing similar assays, we found that INS-18 is processed by KPC-1 at the B-C junction (R51R-R-R54) and by EGL-3 at the C-A junction (K64R65), resulting in a B-A peptide linked by disulfide bonds (Fig. 3B).

In lysates from wild-type animals, the INS-18::GFP reporter migrated more slowly under non-reducing conditions (B-A::GFP) than under reducing conditions (A::GFP) (Fig. 3B, RRKR in the wt lanes, both panels). Under reducing conditions, INS-18::GFP exhibited reduced mobility in lysates from *egl-3* mutants compared with wild-type animals (Fig. 3B, RRKR in the wt and *egl-3* lanes, left panel). Hence, the C-A junction processing requires EGL-3. Importantly, INS-18::GFP exhibited further reduced mobility under non-reducing conditions in *egl-3* mutants (Fig. 3B, RRKR in the *egl-3* lanes, both panels). This indicates that in *egl-3* mutants the B peptide was associated with the non-cleaved C-A::GFP fragment through disulfide bonds, hence the B-C junction was processed. Therefore, EGL-3 is only responsible for cleavage of the C-A junction (Fig. 3C). Indeed, when we mutated this predicted junction (K64R65) to the non-cleavable form, INS-18(A64A65)::GFP exhibited the same mobility as the wild-type reporter in *egl-3* mutants under reducing conditions (Fig. 3B, left panel, RRKR in *egl-3* lane versus RRA64A65 in wt lane).

The B-C junction of INS-18 harbors five tandem arginine residues (R51 to R55), which makes it a candidate for both PC2 (RR) and PC1 (R-X-X-R). To determine the processing site, we mutated each R to A individually in the INS-18::GFP reporter, and found that only R51A and R54A prevented B-C junction processing (data shown for A51-R-R-A54 only, below). The B-C junction R51-X-X-R54 hence resembles a consensus PC1 site: under reducing conditions, INS-18(A51A54)::GFP exhibited an identical migration pattern as the wild-type INS-18::GFP, consistent with the C-A junction being processed in both reporters (Fig. 3C, RRKR and A51A54KR in wt lanes, left panel). Under non-reducing conditions, however, INS-18(A51A54)::GFP exhibited reduced mobility compared with the wild-type reporter, in both wild type and *egl-3* mutants (Fig. 3C, RRKR and A51A54KR in wt and *egl-3* lanes, right panel), consistent with the retention of C peptide in INS-18(A51A54)::GFP.

We confirmed that the B-C junction is processed by KPC-1 by comparing the mobility of the INS-18::GFP reporter in lysates from wild-type and *kpc-1* animals. Under non-reducing conditions, INS-18::GFP exhibited reduced mobility in *kpc-1* mutant lysate because the B-C peptide was associated with A::GFP through disulfide bonds, whereas in lysates from wild-type animals B-C junction processing resulted in the B peptide alone being covalently associated with A::GFP (Fig. 3B, right panel). When disulfide bonds were removed under reducing conditions, INS-18::GFP exhibited the same mobility in wild-type and *kpc-1* animals because only A::GFP was detected (Fig. 3B, left panel).

Mutations in other PCs, namely BLI-4 and AEX-5, had no effect on our wild-type or mutated ILP reporters (Fig. 2A,B; data not shown). The third class of ILPs do not exhibit mobility shift in any PC mutants (Hung et al*.*, 2013; data not shown). Therefore, KPC-1 and EGL-3 mediate the processing of all ILPs examined. Crucially, both PCs are required for the maturation of both antagonistic and agonistic ILPs (Fig. 2B-D, Fig. 3C).

### KPC-1 and EGL-3 regulate dauer formation

As KPC-1 and EGL-3 process the examined ILPs, if these processing events are necessary for ILP functional maturation then KPC-1 and EGL-3 should regulate dauer formation.

We first examined all PC *lf* single mutants. None exhibited obvious *Daf-c* (Table 1). *bli-4;egl-3* and *aex-5;egl-3* double mutants did not exhibit *Daf-c* either (Table 1). Approximately 90% of *kpc-1;egl-3* were embryonic lethal; escapers arrested as sickly early L2 or L3 larvae, but not *Daf-c* (Table 1). Processed ILPs might thus constitute key regulators of embryonic and larval development.

The lack of *Daf-c* in *egl-3* and *kpc-1* single mutants raises two possibilities. First, ILPs that require processing, although essential for development, do not regulate dauer formation. This seems unlikely given the full penetrance of *Daf-c* in *hpDf761;daf-28* mutants. Second, since KPC-1 and EGL-3 contribute redundantly to the maturation of both agonistic and antagonistic ILPs, their removal, while reducing the absolute amount of functional ILPs, does not alter the antagonistic and agonistic input balance to result in *Daf-c*.

The second scenario predicts that a shift in the agonist and antagonistic input ratio is crucial to initiate dauer formation. If this were the case, then although removing either PC is insufficient to induce *Daf-c* on their own, they should modify *Daf-c* penetrance in a sensitized background, such as *lf* mutants for functionally redundant ILPs. Indeed, whereas *daf-28(lf)* exhibited ∼3% *Daf-c*, *egl-3 daf-28(lf)* and *kpc-1;daf-28(lf)* exhibited ∼80% and ∼30% *Daf-c*, respectively (Table 1). The higher *Daf-c* penetrance in *egl-3 daf-28(lf)* is consistent with our finding that the other main dauer-inhibitory agonists, INS-4 and INS-6, are processed by EGL-3. The modest enhancement in *kpc-1;daf-28(lf)* reflects a minor, but physiological, contribution of additional KPC-1 targets. The slightly, but consistently, lower *Daf-c* in *egl-3 daf-28* (∼80%) compared with *hpDf761;daf-28(lf)* (100%) might result from a simultaneous loss of antagonistic INS-1 and INS-18 in the absence of EGL-3.

Consistent with EGL-3 being the processing enzyme for the agonistic ILPs removed by *hpDf761*, *egl-3* did not enhance *hpDf761 Daf-c* penetrance (Table 1). *kpc-1;hpDf761* also exhibited a phenotype similar to that of *kpc-1;egl-3*: ∼80% *kpc-1;hpDf761* died as embryos; escapers arrested as larvae and occasionally sterile adults (Table 1). These genetic interactions indicate that the inhibitory effect of EGL-3 on dauer formation is mainly through processing INS-4 and INS-6.

The high *Daf-c* penetrance of *egl-3 daf-28(lf)* and *hpDf761;daf-28(lf)* mutants indicates that processed ILPs constitute the main activators of insulin signaling to prevent constitutive dauer formation. Since the small population of *kpc-1;egl-3* and *kpc-1;hpDf761* escapers did not activate dauer formation, non-processed ILPs may provide a minor agonistic input for insulin signaling.

### Agonistic ILPs function through sensory or motor neurons

**Fig. 4.**
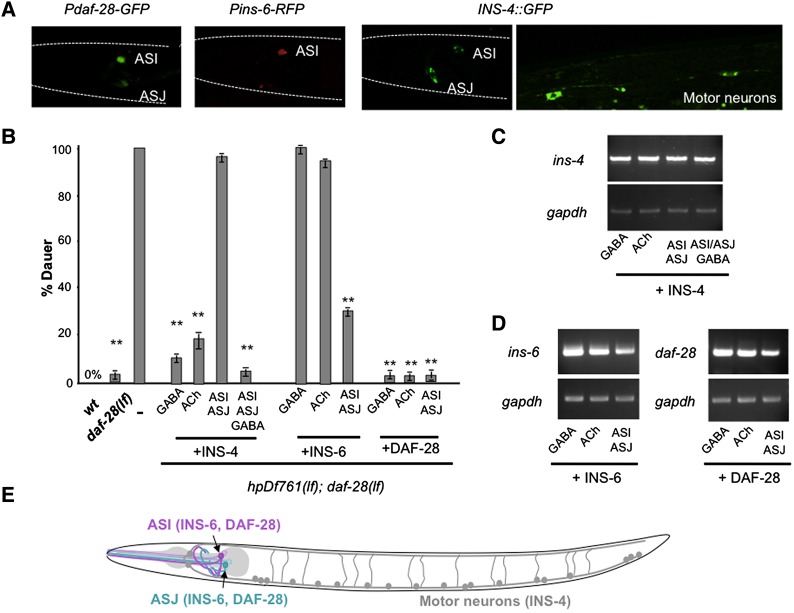
**Agonistic ILPs regulate dauer formation from different neuron groups.** (A) The expression pattern of transcriptional or translational reporters for DAF-28, INS-6 and INS-4. (B) *Daf-c* penetrance of wild-type, *daf-28(lf)*, *hpDf761;daf-28(lf)* and *hpDf761;daf-28(lf)* animals that express INS-4, INS-6 or DAF-28 from different neuronal groups. (C,D) Semi-quantitative RT-PCR analysis of *ins-4*, *ins-6* and *daf-28* from transgenic arrays shown in B. *gapdh* (*gpd-2*) is a loading control. (E) Summary of the physiological origin of agonistic ILPs. **P<0.01 by the Tukey-Kramer comparison test. Error bars, s.d. *N*>150.

We next determined the *C. elegans* tissues that express and are functionally required for ILPs to regulate dauer formation. The expression patterns of INS-4, INS-6 and DAF-28 were examined using both transcriptional and translational reporters. They exhibited substantial, but incomplete, overlap. Consistent with previous reports (Li et al., 2003; Cornils et al., 2011; Hung et al., 2013), *daf-28*, *ins-6* and *ins-4* reporters all exhibited robust expression in the ASI and/or ASJ sensory neurons (Fig. 4A). *ins-4* alone was also expressed weakly and sporadically in ventral cord motor neurons (Fig. 4A).

We examined whether INS-4, INS-6 and DAF-28 function in restricted sets of neurons. For INS-4, we compared the effect of restoring its expression on reversing *Daf-c* of *hpDf761;daf-28(lf)* using neuronal subtype-specific exogenous promoters: from the sensory neurons ASI/ASJ (*Pdaf-28*) alone, GABAergic or cholinergic motor neurons (*Punc-25* or *Pacr-2*) alone, or both sensory and motor neurons (*Pdaf-28+Punc-25*). A combination of sensory and motor neuron-expressed INS-4 fully reverted *Daf-c* penetrance of *hpDf761(lf);daf-28(lf)* from 100% to 6.1% (Fig. 4B, +INS-4 panel, ASI/ASJ/GABA). This suppression was as efficient as restoring INS-4 expression by its endogenous promoter (not shown). Intriguingly, *Pdaf-28*-driven INS-4 alone did not lead to reversion (Fig. 4B, +INS-4 panel, ASI/ASJ). By contrast, expressing INS-4 from GABAergic (*Punc-25*) or cholinergic (*Pacr-2*) motor neurons alone significantly suppressed *Daf-c* (from 100% to 9.7% or 17.7%, respectively; Fig. 4B, +INS-4 panel, GABA, ACh). These results suggest that, despite its weak expression, motor neurons provide a crucial source of INS-4 for dauer regulation.

INS-6 expression was restricted to the ASI and ASJ sensory neurons (Cornils et al., 2011; Hung et al., 2013) (Fig. 4A). Restored INS-6 expression in ASI and ASJ by an exogenous promoter (*Pdaf-28*) reverted the *Daf-c* penetrance of *hpDf761;daf-28(lf)* (100% to ∼30%; Fig. 4B, +INS-6 panel, ASI/ASJ) to a similar degree as from the *ins-6* endogenous promoter (data not shown). Exogenously supplied INS-6 from motor neurons (*Punc-25* or *Pacr-2*), despite abundant expression (Fig. 4C,D), did not alter *Daf-c* penetrance in *hpDf761(lf);daf-28(lf)* (Fig. 4B, +INS-6 panel, GABA, ACh).

As reported (Li et al., 2003; Cornils et al., 2011), DAF-28 was expressed (Fig. 4A) and functionally required (Fig. 4B, +DAF-28 panel, ASI/ASJ) in ASI/ASJ neurons. Unlike INS-6, however, DAF-28 could function equally efficiently in reverting the *Daf-c* penetrance of *hpDf761;daf-28(lf)* when ectopically supplied from motor neurons (Fig. 4B, +DAF-28 panel, +GABA, +ACh).

These results reveal that the main agonistic ligands function from sensory and motor neurons, and their redundancy is not straightforward: whereas DAF-28 functions redundantly with, and can substitute for, either INS-4 or INS-6 in their respective neurons of origin, INS-4 and INS-6 function from different neuronal groups and cannot replace each other. These results suggest that DAF-28 might represent a stronger ligand than INS-4 or INS-6. This is consistent with the observation that DAF-28(gf) from ASI and ASJ neurons, both residing anteriorly, can block DAF-2 activity throughout the body (see Discussion).

### Antagonistic ILPs are also likely to function through neurons

**Fig. 5.**
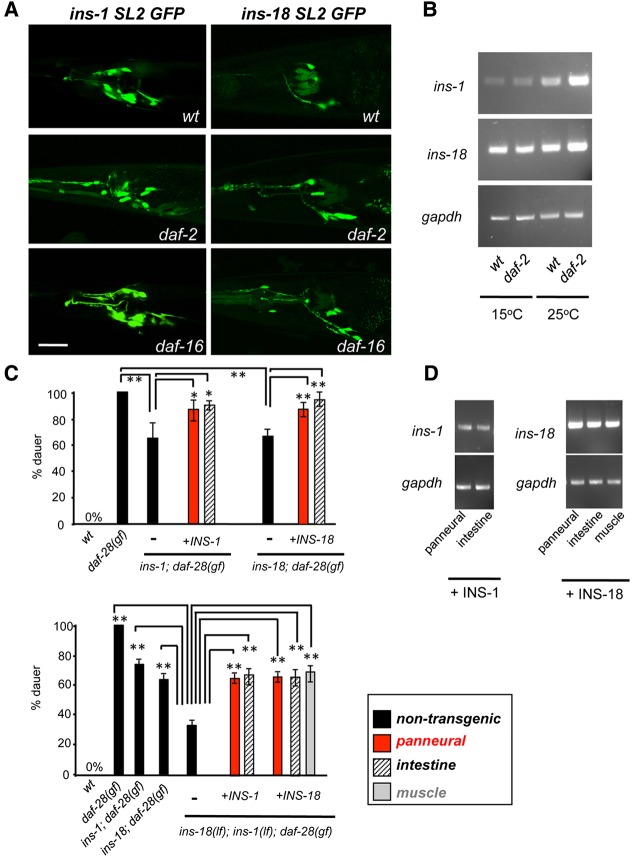
**Antagonistic ILPs are expressed by neurons.** (A) Expression patterns of INS-1 or INS-18 fosmid bi-cistronic GFP reporter in wild-type animals, *daf-2(ts)* and *daf-16* mutants. (B) *ins-1* and *ins-18* transcripts in wild-type and *daf-2* mutants at permissive (15°C) and non-permissive (25°C) temperature with *gapdh* as the loading control. (C) *Daf-c* penetrance in wild-type, *daf-28(gf)*, *ins-18;daf-28(gf)*, *ins-1;daf-28(gf)* and *ins-18;ins-1;daf-28(gf)* animals, expressing INS-1 or INS-18 from a pan-neural, intestine or muscle promoter. Expression of INS-18 and INS-1 in any tissue led to a partial reversion of *Daf-c* penetrance. **P<0.01, *P<0.05, with the Tukey-Kramer comparison test. Error bars, s.d. *N*>150. (D) Semi-quantitative RT-PCR analysis of transgenic lines in B. Scale bar: 5 µm.

Transcriptional reporters for *ins-1* and *ins-18* are active in many sensory neurons, motor neurons and the intestine (Pierce et al., 2001). To examine the expression of antagonistic ILPs in a more endogenous genomic context, we generated functional fosmid INS-18 and INS-1 reporters. These reporters exhibited more restricted expression patterns, with strong sensory neuron expression (Fig. 5A, wt panels). Neither reporter exhibited intestinal expression.

The lack of intestinal expression suggests that either INS-1 and INS-18, like agonistic ILPs, are also provided by the nervous system or their intestinal expression is dynamically regulated during dauer formation. To test the second possibility, we examined these reporters in *daf-2* and *daf-16* mutants. We did not observe intestinal activation during any stage of dauer formation in either mutant (Fig. 5A).

Pan-neural restoration of either ILP led to significant reversion of *Daf-c* penetrance in the respective *ins;daf-28(gf;ts)* mutants (from ∼60% to ∼85%; Fig. 5C, top panel). We further tested the effect of co-restoring their expression in *ins-18;ins-1;daf-28(gf;ts)* triple mutants, which exhibit a more significant reduction of *Daf-c* (from 100% to ∼30%). We again observed significant reversion of *Daf-c* (from ∼30% to ∼60%; Fig. 5C, bottom panel, hatched bars). The partial, but significant, reversion was not due to insufficient expression (Fig. 5D; data not shown). One possibility is that an overexpression of antagonistic ILPs might have weakly enhanced *Daf-c* in *daf-28(gf;ts)*, as is the case for *daf-2* weak alleles (Pierce et al., 2001; Cornils et al., 2011). Together, these results led us to favor the possibility that physiological antagonistic ILPs are also provided by the nervous system to promote dauer formation.

INS-1 and INS-18 are functional regardless of their cellular origin. At similar expression levels (Fig. 5D), not only neuronally but also intestinally expressed INS-1 and INS-18 exhibited similar rescue efficiency in *ins-1;ins-18;daf-28(gf)* (Fig. 5C), and INS-18::RFP expressed from muscles also rescued to a similar extent (Fig. 5C, gray bar).

### ILP processing enzymes can function extracellularly

**Fig. 6.**
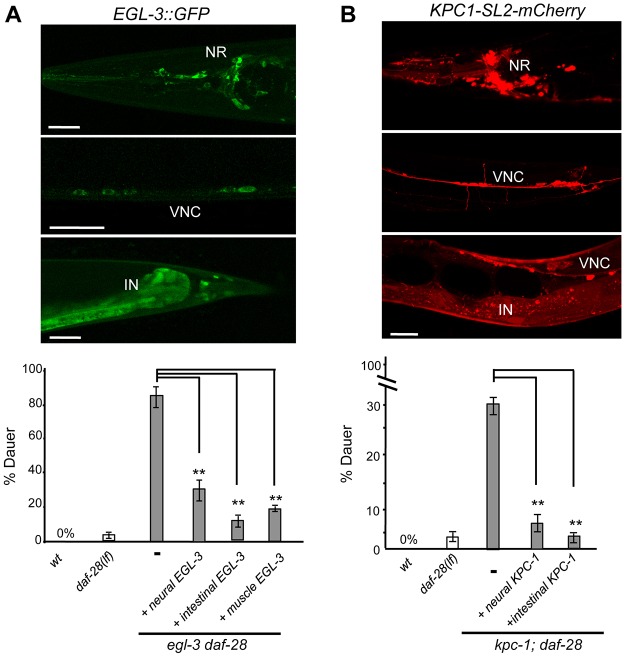
**EGL-3 and KPC-1 are expressed in neurons and intestine.** (A) (Top) The expression pattern of a functional EGL-3::GFP reporter. NR, nerve ring; VNC, ventral nerve cord; IN, intestine. (Bottom) The penetrance of *Daf-c* in wild-type, *daf-28(lf)* and *egl-3 daf-28(lf)* animals, and transgenic *egl-3 daf-28(lf)* animals expressing EGL-3 either pan-neurally, in the intestine, or in muscle. (B) (Top) A functional, bi-cistronic RFP reporter for KPC-1 also exhibits RFP signals in the nervous system and intestine. (Bottom) *Daf-c* penetrance of wild-type, *daf-28(lf)* and *kpc-1;daf-28(lf)* mutants, and transgenic *kpc-1;daf-28(lf)* animals expressing KPC-1 either pan-neurally or in the intestine. **P<0.01 by the Tukey-Kramer comparison test. Error bars, s.d. *N*>150. Scale bars: 5 µm.

Consistent with a neuronal origin of dauer-regulating ILPs, their processing enzymes, EGL-3 and KPC-1, are expressed by the nervous system. Our EGL-3::GFP plasmid reporter, as previously reported (Kass et al., 2001), exhibited broad expression in the nervous system (Fig. 6A, NR and VNC) and the intestine (Fig. 6A, IN). Our functional KPC-1 fosmid reporter also exhibited expression in the nervous system (Fig. 6B, NR and VNC) and intestine (Fig. 6B, IN).

We performed *egl-3* and *kpc-1* rescue experiments using tissue-specific promoters, originally designed to distinguish the functional contribution of neurons and intestine. Unexpectedly, they revealed that ILP processing enzymes can function extracellularly. Restoring EGL-3 expression in either neurons or the intestine in *egl-3 daf-28(lf)* mutants similarly reverted *egl-3* enhancement of *Daf-c* penetrance (from ∼80% to ∼31% or ∼13%, respectively; Fig. 6A). Similarly, restoring KPC-1 from either neurons or the intestine reverted the *Daf-c* penetrance of *kpc-1;daf-28(lf)* from ∼28% to ∼6% or ∼3%, respectively (Fig. 6B).

*egl-3* enhanced *daf-28(lf)* mainly through its role in processing INS-4 and INS-6, two ILPs expressed only in neurons. The ability of intestinal EGL-3 to rescue *egl-3 daf-28(lf)* suggests that EGL-3 can be secreted and function extracellularly to process ILPs. This would explain the ability of exogenous INS-18::RFP from muscles, where EGL-3 is not present, to affect dauer formation (Fig. 5B). Indeed, ectopic expression of EGL-3 by a muscle-specific promoter also led to partial rescue of *egl-3 daf-28(lf) Daf-c* (Fig. 6A)*.* A previous study noted that another *C. elegans* PC, AEX-5, is functional upon secretion (Mahoney et al., 2008).

### Intestinal insulin signaling activity determines dauer formation

Core components of the *C. elegans* insulin signaling pathway, which is the effector of dauer-regulating ILPs, are expressed ubiquitously. We determined the tissue requirement of DAF-2 and its effector DAF-16 for dauer formation.

We compared the effect of restoring DAF-2 in all somatic tissues (*Pdpy-30*) or specifically in the nervous system (*Prgef-1*), muscles (*Pmyo-3*) or intestine (*Pges-1*) in *daf-2(lf;ts)*. Expression of a DAF-2 mini-gene from the ubiquitous promoter rescued the *Daf-c* phenotype of *daf-2(lf;ts)* from 100% to 0% (Table 2). Restored expression of the same DAF-2 mini-gene in the intestine alone rescued *daf-2 Daf-c* penetrance as effectively (Table 2). Expression of this mini-gene in neurons, muscles, or neurons plus muscles, did not result in any rescue (100%, Table 2). Therefore, ILPs converge on the intestinal InR to regulate the choice between reproductive growth and diapause.

**Table 2.**
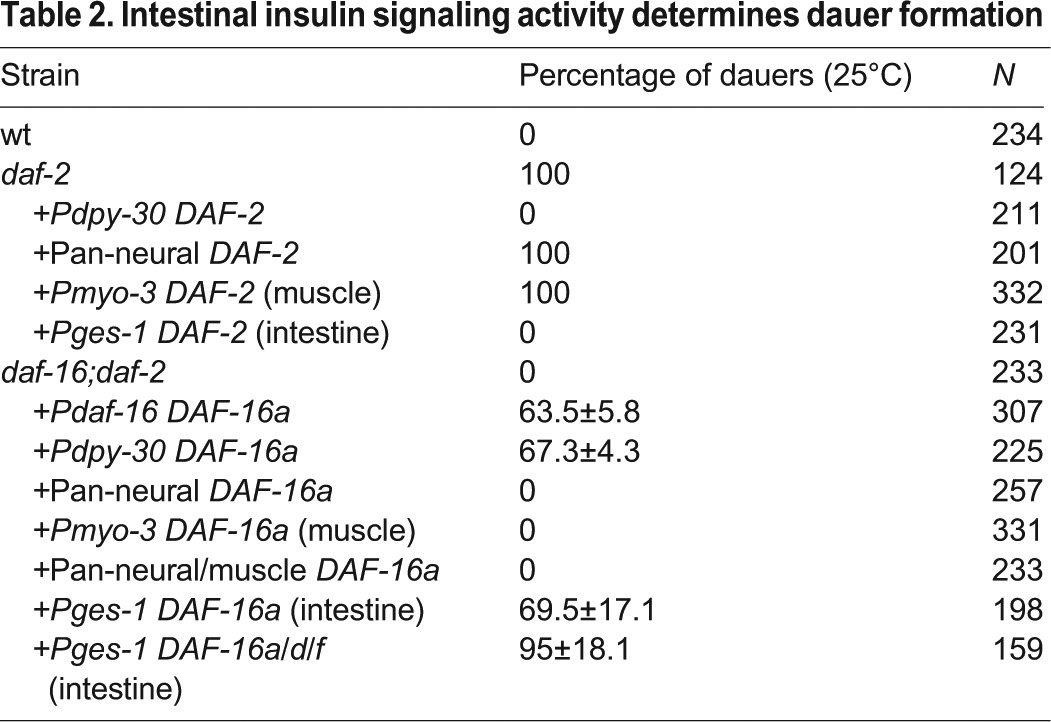
**Intestinal insulin signaling activity determines dauer formation**

We next examined the tissue requirement of DAF-16, the main effector of DAF-2. The *daf-16* locus encodes multiple DAF-16 isoforms (Ogg et al., 1997; Libina et al., 2003; Kwon et al., 2010). *daf-16(lf;null);daf-2(lf;ts)* exhibited 0% *Daf-c* (Libina et al., 2003; Kwon et al., 2010). Restoring the expression of a DAF-16 isoform, DAF-16a, under its endogenous promoter, reverted the *Daf-c* of *daf-16;daf-2* from 0% to ∼60% (supplementary material Fig. S2B). Restoring DAF-16a either in all tissues (*Pdpy-30*) or in the intestine alone (*Pges-1*) reverted *Daf-c* penetrance to essentially the same extent, from 0% to ∼67% or ∼70%, respectively (Table 2), whereas expression of DAF-16a in muscles or neurons had no effect (0%) (Table 2). All tissue-specific transgenes exhibited nuclear localization of DAF-16a in *daf-2(lf;ts)* mutants (supplementary material Fig. S3B). Hence, the lack of rescue could not be attributed to a tissue-specific regulation of DAF-16a function.

The high but incomplete rescue by DAF-16a in *daf-16;daf-2* is due to a functional requirement of another DAF-16 isoform, DAF-16d/f. We examined the effect of transgenes that specifically express DAF-16a, DAF-16b or DAF-16d/f under their endogenous promoters (Kwon et al., 2010) in *daf-16;daf-2*. Both DAF-16a and DAF-16d/f partially reverted the *Daf-c* penetrance, whereas DAF-16b did not (supplementary material Fig. S2B). Co-expression of DAF-16a and DAF-16d/f in the intestine of *daf-16;daf-2* mutants led to full *Daf-c* penetrance (Table 2). Therefore, both DAF-2 and DAF-16 function through the intestine to regulate dauer formation.

**Fig. 7.**
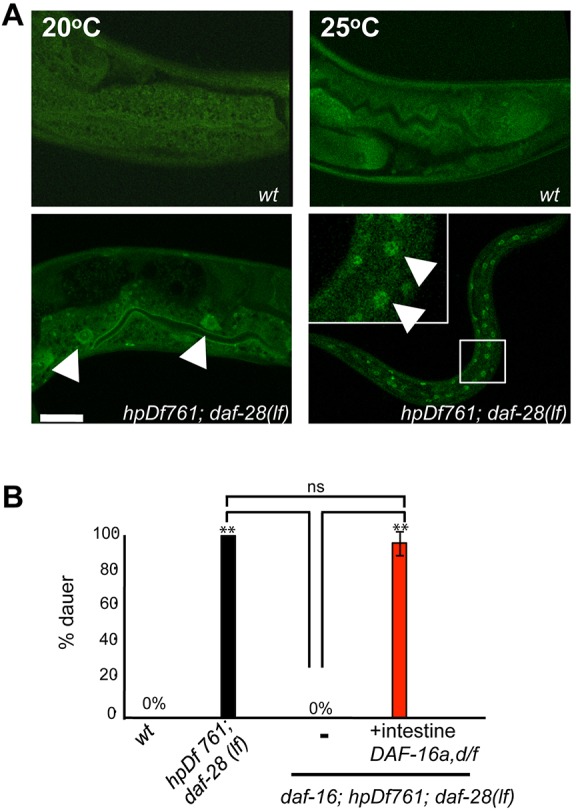
**DAF-16a::GFP localizes to the nucleus in the absence of agonistic ILPs.** (A) Representative images of a functional DAF-16a::GFP reporter in fixed animals. (Top) DAF-16a::GFP exhibits cytoplasmic expression regardless of temperature in a wild-type background. (Bottom) In *hpDf761;daf-28(lf)* animals, DAF-16a::GFP translocates to some or to all intestinal nuclei (arrowheads) at semi-permissive (20°C) or non-permissive (25°C) temperatures, respectively. (B) *daf-16* suppresses the *Daf-c* of *hpDf761;daf-28(lf)*. This suppression is reverted by co-expressing DAF-16a and DAF-16d/f in the intestine. **P<0.01 by the Tukey-Kramer comparison test. Error bars, s.d. *N*>150. Scale bar: 5 µm.

The loss of DAF-16 fully inhibited the *Daf-c* phenotype of the ILP mutants *hpDf761;daf-28(lf)* and *egl-3 daf-28(lf)* (Table 2). As in *daf-2* mutants, intestinal DAF-16a::GFP became nuclear localized in *hpDf761;daf-28(lf)* animals at non-permissive temperatures (Fig. 7A). Crucially, restored co-expression of DAF-16a and DAF-16 d/f in the intestine also fully reverted the *Daf-c* penetrance of *daf-16;hpDf761;daf-28(lf)* (Fig. 7B). Hence, neuronal ILPs converge on the intestinal insulin signaling activity to regulate reproductive development versus dauer formation.

## DISCUSSION

**Fig. 8.**
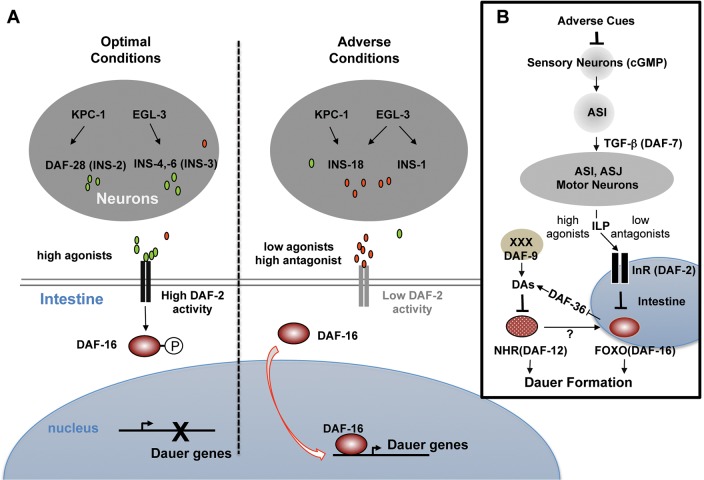
**Neuron-intestine communication determines the developmental program.** (A) Neuronal ILPs determine the state of intestinal insulin signaling activity. Under optimal conditions, the nervous system establishes a high agonist/low antagonist paradigm to maintain high intestinal DAF-2 and inhibit DAF-16. Adverse conditions induce a reduction of agonists, permitting increased antagonists to activate DAF-16, committing animals for dauer formation. (B) A proposed sequential signaling model for developmental decision. Cues that signal environmental adversity are sensed by sensory neurons, which triggers reduced DAF-7/TGFβ secretion from the ASI sensory neuron. This leads to decreased agonistic and increased antagonistic ILPs, and the activation of intestinal DAF-16. DAF-16 reduces the synthesis of DA from the intestine and XXX cells, resulting in unliganded DAF-12 that reciprocally activates DAF-16. Unliganded DAF-12 and intestinal DAF-16 initiate the transcriptional programs that underlie tissue and metabolic remodeling in dauers.

In the present study, we show that multiple ILPs, processed by EGL-3 and/or KPC-1, regulate insulin signaling in the intestine to determine the choice between the dauer and reproductive programs. We propose the following model (Fig. 8A). Under normal conditions, the nervous system employs multiple agonistic ligands, namely INS-4, INS-6 and DAF-28, from the sensory and motor neurons to maintain high intestinal DAF-2 activity and sequester intestinal DAF-16, which prevents dauer formation. Under adverse conditions, the nervous system orchestrates a reduction of agonistic and an increase of antagonistic inputs to decrease intestinal DAF-2 activity. The subsequent activation of intestinal DAF-16 turns on the transcriptional network that initiates and underlies dauer development.

### An ILP code for dauer decision: from multiple neurons and a sequential effect

Expanding on previous studies (Cornils et al., 2011; Pierce et al., 2001; Li et al., 2003), we determined a cohort of key ILPs that regulate dauer formation. The agonistic ILPs DAF-28, INS-4 and INS-6 are expressed by, and functionally sufficient from, the sensory or motor neurons. The partial overlap of their expression patterns and neuronal requirement suggests that DAF-28 functions redundantly with INS-6 as the long-range ligands and with INS-4 as the short-range ligands that activate intestinal DAF-2 receptors. DAF-28 and INS-6 also exhibit functional differences in preventing dauer entry and promoting dauer exit, respectively (Cornils et al., 2011).

*daf-28(gf)* was speculated to cause *Daf-c* by non-specifically preventing other ILP processing (Li et al., 2003). This would be unlikely if INS-4 and INS-6/DAF-28 were to function from separate neuronal groups. Using the ILP processing assay, we observed that the INS-4::GFP reporter, expressed either pan-neurally or from DAF-28-producing neurons, was processed in *daf-28(gf)* mutants (supplementary material Fig. S4). The observation that DAF-28, when secreted from anterior sensory neurons alone, could functionally compensate for both INS-6 and INS-4, also suggests that DAF-28 might be a preferred DAF-2 ligand. We propose that DAF-28(gf), as an inactive but high-affinity ligand for DAF-2, blocks other agonistic ligands through stereo-hindrance.

Antagonistic ILPs are also likely to function through neurons. *daf-2* and *daf-16* mutants exhibit increased and decreased *ins-18* expression, respectively (Murphy et al., 2003). We observed an increase of both *ins-1* and *ins-18* transcripts in *daf-2* mutants (Fig. 5B). Whereas constitutive overexpression of INS-1 or INS-18 from either neurons or the intestine drastically increased the *Daf-c* penetrance of *daf-2(lf;ts)*, their overexpression was inefficient in inducing *Daf-c* in a wild-type background (data not shown). Increasing antagonistic ligands alone is thus insufficient to initiate dauer formation; this implies that antagonistic ILPs are unable to access InR/DAF-2 in the presence of agonistic ILPs.

We propose that, in addition to a balance between agonistic and antagonistic ILPs, the prerequisite to initiate dauer formation should be a reduction of agonistic ILPs that maintain, by default, high intestinal insulin signaling activity to ensure reproductive development. Only upon the reduction of agonistic ILPs, can antagonistic ILPs efficiently activate intestinal DAF-16 to initiate dauer formation.

### EGL-3 and KPC-1 are *C. elegans* PC2 and PC1

All examined *C. elegans* agonists, i.e. INS-3, INS-4, INS-6 and DAF-28, belong to the β group, for which F peptide processing is mediated by either KPC-1 or EGL-3. INS-7, which mildly enhances the *Daf-c* penetrance of a weak *daf-2(lf)* allele (Murphy et al., 2003), might function as another minor agonist. It also belongs to the β class, with an EGL-3-like processing junction.

Antagonistic ligands constitute the α class. EGL-3 alone (INS-1) or both EGL-3 and KPC-1 (INS-18) remove their C peptides. In all cases, EGL-3 processes the RR/KR and KPC-1 the R-X-X-R site. Hence, KPC-1 and EGL-3 represent PC1 and PC2 for the examined ILPs. From a structural aspect, INS-18 represents the closest homolog of mammalian insulin.

The crucial role of EGL-3 and KPC-1 in dauer formation is masked by their involvement in the maturation of both agonistic and agonistic ILPs. EGL-3 and KPC-1 also process other neuropeptides (Husson et al., 2006). We could not exclude the possibility that the embryonic lethality in *kpc-1;egl-3* and *kpc-1;hpDf761* mutants results from not only the loss of processed ILPs, but also the loss of small neuropeptides. For dauer regulation, however, the *Daf-c* penetrance of *egl-3 daf-28(lf)* mutants argues strongly for the loss of EGL-3-dependent ILPs being the main cause of constitutive dauer formation.

### Intestinal insulin signaling determines dauer versus reproductive development

Both InR/DAF-2 and its effector FOXO/DAF-16 are required exclusively at the intestine to regulate dauer formation; hence, insulin signaling activity in the intestine dictates the choice of developmental programs. DAF-16 functions through the intestine to regulate longevity (Libina et al., 2003; Murphy et al., 2007). We propose that the intestine is the signaling center to determine both developmental strategies and longevity.

These conclusions differ from those of previous studies, which proposed that DAF-2 and DAF-16 function from multiple tissues, mainly the nervous system, to regulate dauer formation (Apfeld and Kenyon, 1998; Wolkow et al., 2000). This discrepancy might result from differences in experimental approaches and reagents. By mosaic analyses, DAF-2 was shown to affect dauer formation through multiple lineages, predominantly those that give rise to the nervous system (Apfeld and Kenyon, 1998). The loss or presence of DAF-2 in the intestine, however, could not be directly determined by the lineage marker (NCL-1) used in this study; hence, the effect was inferred from the phenotype of mosaic animals in other lineages (Apfeld and Kenyon, 1998). Results from DAF-2 tissue-specific rescue experiments (Wolkow et al., 2000) led to consistent, but not unambiguous, conclusions: DAF-2 driven by two pan-neural promoters showed strong (*Punc-14*) or partial (*Punc-119*) rescue of *daf-2 Daf-c*, whereas DAF-2 driven by an intestinal promoter had no effect. The authors noted that *Punc-14* exhibited leaky expression in the pharynx and/or intestine (footnote 23 in Wolkow et al., 2000). The requirement of DAF-16 was examined by both tissue-specific rescue experiments and mosaic analyses. The mosaic analyses suggest that DAF-16 might affect dauer state from multiple lineages (Libina et al., 2003). Similar to the case with DAF-2, neuronally expressed DAF-16 led to a partial rescue of dauer regulation, whereas the intestinal restoration had no effect (Libina et al., 2003). We speculate that differences in the expression constructs (promoters and cDNAs) led to different results in these studies.

*C. elegans* insulin signaling regulates multiple biological processes. A key question is how a single InR achieves functional specificity. There are multiple potential mechanisms: DAF-2 has an inherent differential affinity for different ILPs; local ILP cohorts fine-tune DAF-2 activity in different cells; DAF-16 activates tissue- and developmental stage-specific targets. The necessity and sufficiency of intestinal DAF-16 in preventing diapause indicate that intestine-specific DAF-16 targets hold the key to developmental programs.

DAF-12 functions genetically downstream of DAF-16. DAF-36, a synthesizing enzyme for the more potent DAF-12 ligand Δ^7^-DA, and the DAF-36 positive regulator NHR-8 are both expressed by the intestine (Rottiers et al., 2006; Magner et al., 2013). *daf-16* mutants exhibit a drastic increase of DAF-36 metabolic products (Magner et al., 2013). Intestinal DAF-16 might initiate diapause in part through reducing DA synthesis, leading to dauer-promoting DAF-12 activity (see below).

### Interplay between insulin signaling and other regulators

Insulin signaling is but one of several pathways that affect the dauer decision. Both cGMP signaling in multiple sensory neurons (Birnby et al., 2000) and TGFβ (DAF-7) signaling through the ASI sensory neuron (Ren et al., 1996; Schackwitz et al., 1996) function genetically upstream of DAF-16 (Larsen et al., 1995; Lee et al., 2001b). Other regulators, such as the steroid DAs and their receptor DAF-12 (Antebi et al., 2000; Jia et al., 2002; Motola et al., 2006), function genetically downstream of the TGFβ signaling but exhibit complex interactions with DAF-16. DA-bound DAF-12 promotes reproductive development, whereas unliganded DAF-12 is required for dauer formation (reviewed by Antebi, 2013). DAF-9, which is the enzyme for the last step in DA synthesis (Gerisch et al., 2001; Jia et al., 2002), and DAF-12 function genetically downstream of DAF-16. However, they affect each other's expression, forming a circular transcriptional regulation loop (Jeong et al., 2010). In addition, DAF-16 negatively regulates a DA precursor generated by DAF-36, a Δ^7^-DA-synthesizing enzyme expressed in the intestine (Magner et al., 2013).

Based on these interactions and the involvement of several signaling components (reviewed by Hu, 2007; Antebi, 2013), we postulate a sequential model for dauer formation (Fig. 8B). An adverse cue is registered by sensory neurons, which triggers a reduction in TGFβ secretion from the ASI sensory neuron. This initiates the reduction of agonistic ILP from sensory and motor neurons, which normally maintains a high intestinal DAF-2 activity to prevent DAF-16 activation. Reduced agonistic and increased antagonistic ILPs lead to DAF-16 activation in the intestine. Intestinal DAF-16 switches DAF-12 activity from promoting reproductive development to dauer formation, through reducing the synthesis of its steroid DA ligands. Reduced DA triggers a feed-forward activation loop for unliganded DAF-12 and DAF-16. Together, they initiate transcriptional changes that underlie diapause.

Several questions related to this model remain to be addressed. First, three key sensory neurons that prevent *Daf-c*, namely ASI, ASJ and ADF, were identified by cell ablation studies. Agonistic ILPs and TGFβ are secreted by ASI and/or ASJ, providing underlying mechanisms for their inhibition of dauer formation. ADF ablation leads to a similar degree of *Daf-c* as ablating ASI (Bargmann and Horvitz, 1991), but mechanisms that underlie the role of ADF are unknown. Unidentified signaling molecules might function in the ADF; alternatively, ADF might potentiate ASI- and ASJ-mediated secretion of agonistic ILPs and TGFβ.

Second, recent studies suggest that TGFβ and insulin signaling converge their regulation on ILP expression and, subsequently, on DAF-16 activity (Liu et al., 2004; Narasimhan et al., 2011). This raises the possibility that decreased TGFβ secretion from ASI might initiate the reduction in agonistic ILPs. If this were the case, then TGFβ receptors (DAF-1, DAF-4) and effectors (DAF-3, DAF-8, DAF-14) that regulate dauer decision should function in relevant ILP-producing sensory and motor neurons.

Third, the dauer state requires the activation of DAF-16 and unliganded DAF-12. The endocrine-like XXX cell-derived DAs (Schaedel et al., 2012), synthesized either directly by DAF-9 (expressed by XXX) or by the sequential enzymatic reactions, from DAF-36 (expressed by intestine) to DAF-9 (expressed by XXX) (Rottiers et al., 2006), activate DAF-12 to promote reproductive development (Gerisch et al., 2001; Jia et al., 2002; Motola et al., 2006). Does DAF-16 initiate changes in DAF-12 activity by affecting the DA composition? If so, does intestinal DAF-16 initiate dauer formation in part through reducing DAF-36 expression? How does DAF-12 regulate DAF-16 activity? Since intestinal DAF-16 controls both larval development and aging, mechanisms for DA-regulated longevity (reviewed by Antebi, 2013) might provide clues as to its role in dauer formation.

## MATERIALS AND METHODS

### Strains

*C. elegans* were cultured on OP50-seeded NGM plates. Non-*ts* strains were maintained at 22°C and *ts* strains at 16°C. Deletion strains were outcrossed against N2 at least four times. All strains were maintained in homozygous backgrounds except *kpc-1(gk8);egl-3(ok979)* [maintained in *kpc-1(gk8);egl-3(ok979)/hpIs242* balancer] and *kpc-1(gk8);hpDf761* [maintained in *kpc-1(gk8);hpDf761/unc-104(e1265) juIs76* balancer]*.* A strain list is provided in supplementary material Tables S1 and S2.

### Generation of *hpDf761*

*hpDf761* was generated using Mos1-induced homologous recombination (Frokjaer-Jensen et al., 2010, 2012), with pJH2606 (the targeting construct) and *unc-119(ed3);ttTi13603* (gift of Jean-Louis Bessereau). *unc-119* was outcrossed prior to analyses.

### Constructs

A list of constructs is provided in supplementary material Table S2.

### Insulin processing assay

The insulin processing assay was carried out as described previously (Hung et al., 2013).

### Dauer assay

L4 animals, maintained at 22°C (non-*ts*) or 16°C (*ts*), were transferred to new plates (one per plate) at 25°C, and removed 24 h later. The percentage of dauer progeny was scored 48 h afterwards. With non-integrated transgenic lines, dauer frequency was scored in the transgenic population.

### Quantification of DAF-16::GFP signals

Animals carrying integrated DAF-16a::GFP (Lin et al., 2001) were fixed in 5% paraformaldehyde (Hung et al., 2013) to prevent GFP translocation during imaging.

### Semi-quantitative RT-PCR

RNA isolation (from 200 hand-picked transgenic animals) and RT-PCR were performed as previously described (Ramani et al., 2011).

## Supplementary Material

Supplementary Material
