# Supplementary material for: A Caenorhabditis elegans developmental decision requires insulin signaling-mediated neuron-intestine communication
Source: Development. 2014 Apr;141(8):1767–79. doi: 10.1242/dev.103846 (PMC3978837; doi:10.1242/dev.103846)
Supplement: Supplementary Material [file supp_141_8_1767__index.html]

Supplementary Material 

# A *Caenorhabditis elegans* developmental decision requires insulin signaling-mediated neuron-intestine communication

## DEV103846 Supplementary Material

**Files in this Data Supplement:**

- **Supplementary Material**
