# Supplementary material for: A Caenorhabditis elegans developmental decision requires insulin signaling-mediated neuron-intestine communication
Source: Development. 2014 Apr;141(8):1767–79. doi: 10.1242/dev.103846 (PMC3978837; doi:10.1242/dev.103846)
Supplement: Supplementary Material [file supp_dev.103846_DEV103846.pdf]

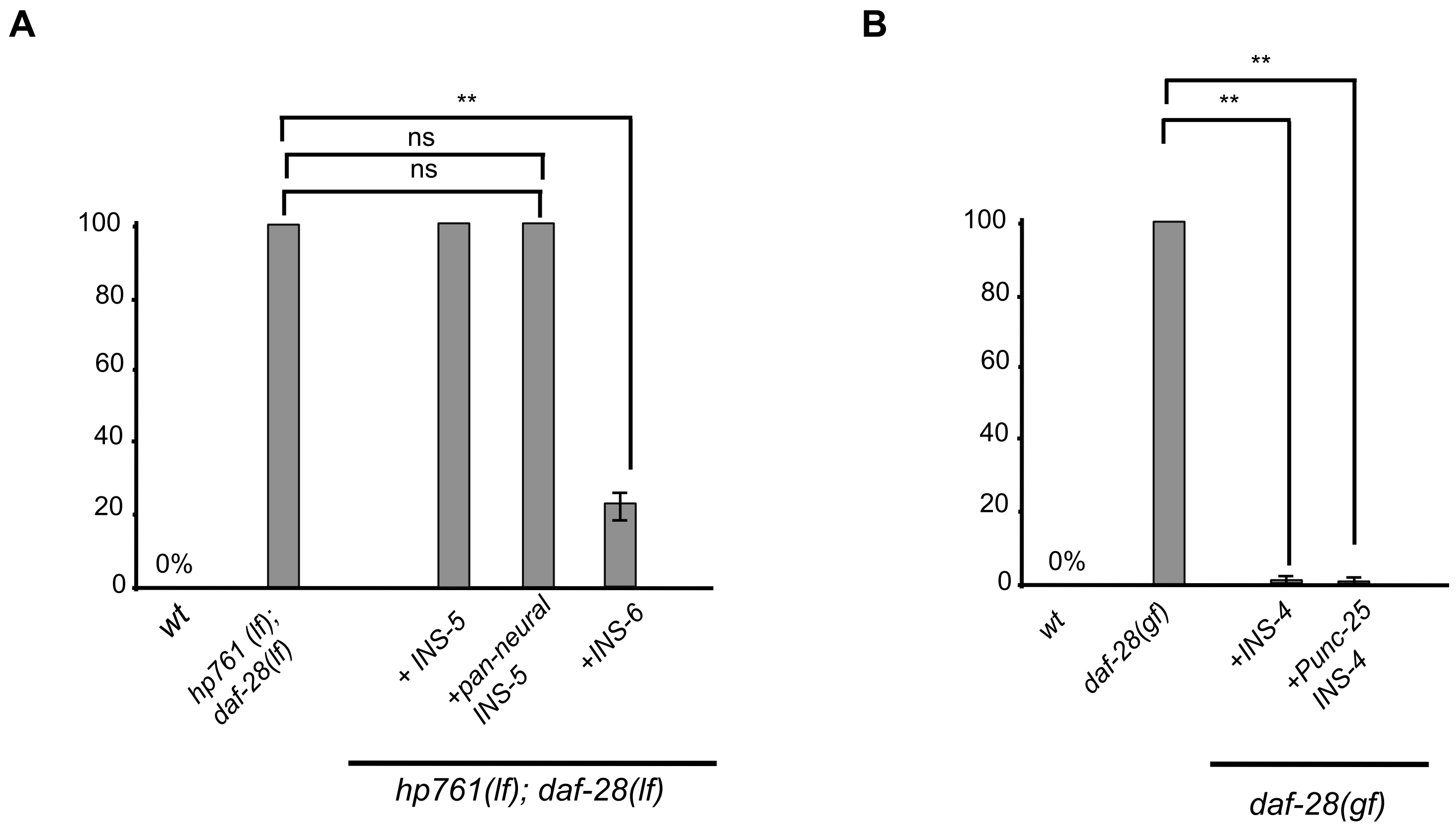
A
**
ns
ns
100
80
60
40
20
0
0%
wt
hp761 (lf); daf-28(lf)
+ INS-5
+pan-neural INS-5
+INS-6
hp761(lf); daf-28(lf)
B
**
**
100
80
60
40
20
0
0%
wt
daf-28(gf)
+INS-4
+Punc-25 INS-4
daf-28(gf)

Figure S1

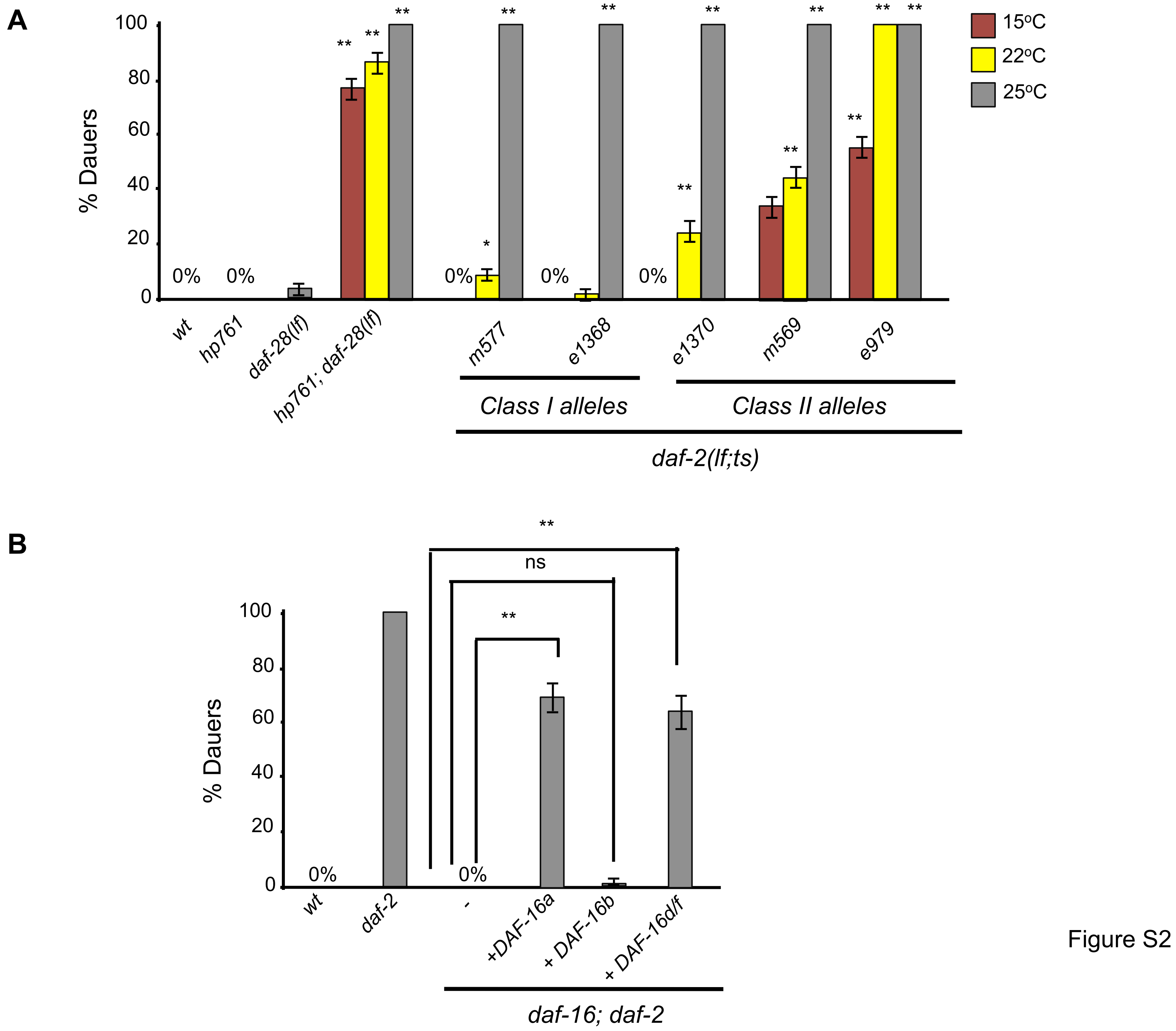

A
B
% Dauers
100
80
60
40
20
0
0%
**
*
15oC
22oC
25oC
wt
hp761
daf-28(lf)
hp761; daf-28(lf)
m577
e1368
e1370
m569
e979
Class I alleles
Class II alleles
daf-2(lf;ts)
ns
daf-2
-
+DAF-16a
+ DAF-16b
+ DAF-16d/f
daf-16; daf-2

Figure S2

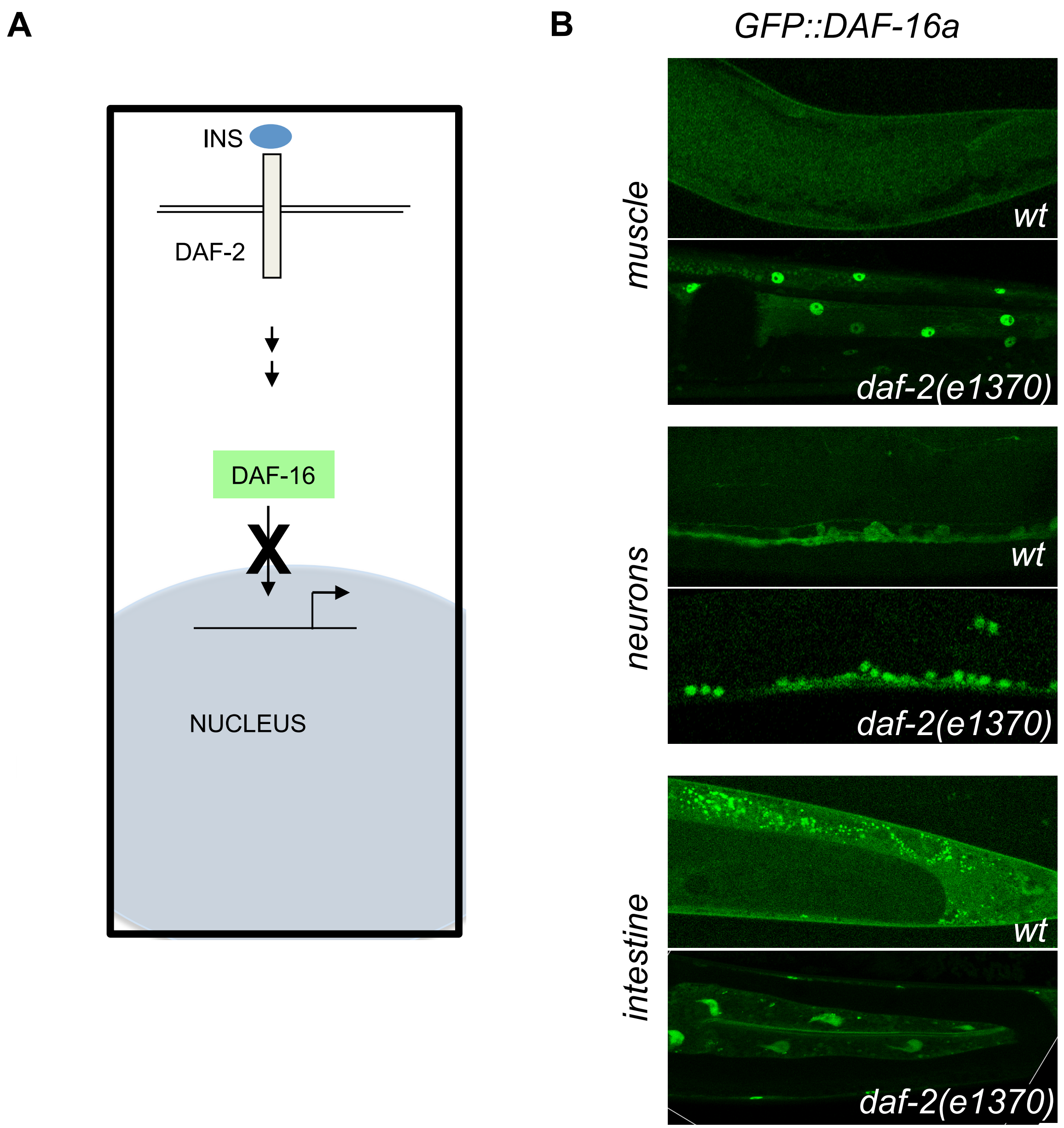
A
INS
DAF-2
DAF-16
X
NUCLEUS
B
GFP::DAF-16a
muscle
wt
daf-2(e1370)
neurons
wt
daf-2(e1370)
intestine
wt
daf-2(e1370)

Figure S3

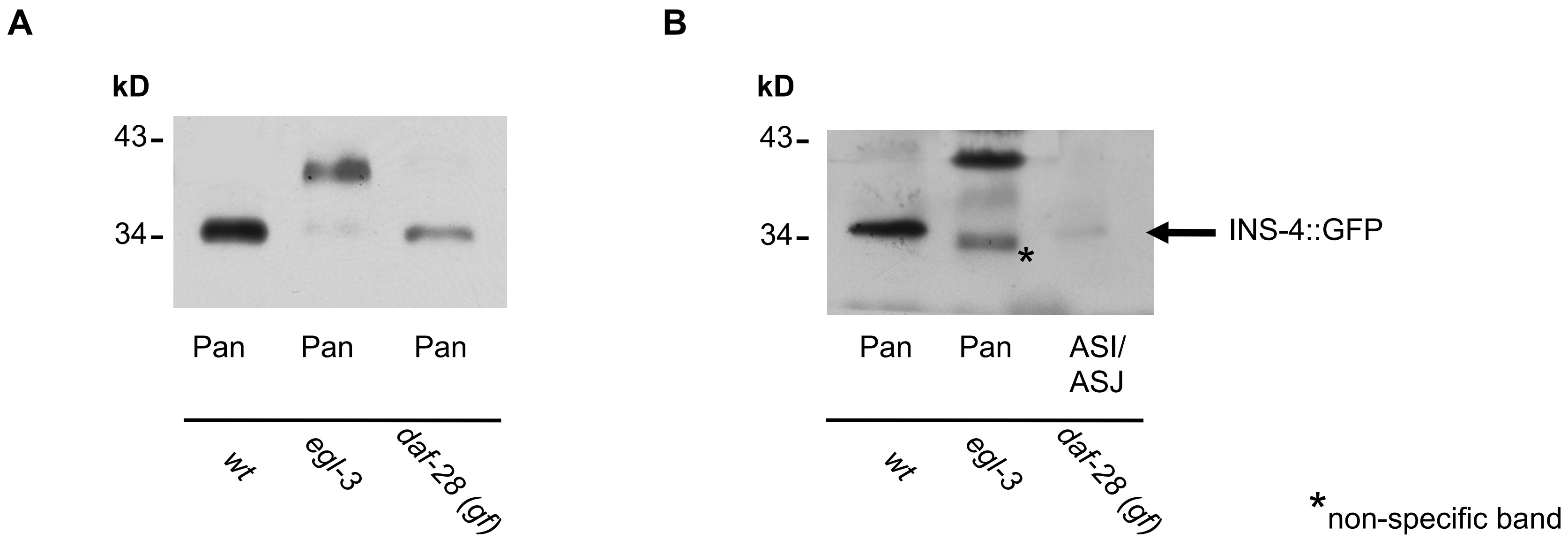
A
B
kD
43
34
Pan
Pan
Pan
wt
egl-3
daf-28 (gf)
kD
43
34
INS-4::GFP
*
Pan
Pan
ASI/
ASJ
wt
egl-3
daf-28 (gf)
*non-specific band

Figure S4

Supplemental Figure Legend

Fig. S1. INS-5 does not regulate dauer formation

(A) Wild-type animals (*wt*) and *hpDf761; daf-28(lf)* mutants exhibit 0% and 100% *Daf-c*, respectively. Transgenic *hpDf761; daf-28(lf)* animals that overexpress INS-5, either from its endogenous promoter (+INS-5), or from an exogenous pan-neuronal promoter (+pan-neural INS-5), fail to alter the *Daf-c* penetrance. Overexpressing INS-6, under its endogenous promoter (+INS-6), in *hp761; daf-28(lf)*, by contrast, led to a significant suppression of *Daf-c* penetrance. (B) An overexpression of INS-4 from either its endogenous promoter (+INS-4), or a GABAergic motor neuron promoter (+Punc-25-INS-4), in *daf-28(gf)* led to a full suppression of its *Daf-c* penetrance. **, $p<0.001$, ns, $p>0.05$ by the Tukey-Kramer comparison test. At least 200 animals were examined in 3 trials. Error bars, standard deviation.

Figure S2.

(A) *hp761; daf-28(lf)* mutants exhibit *Daf-c* penetrance that mimics *daf-2(lf)*

A comparison of *Daf-c* penetrance between wild-type animals (*wt*), *hp761; daf-28(lf)* mutants, and five *daf-2(lf;ts)* alleles, at 15ºC (grey bars), 22ºC (yellow bars) and 25ºC (red bars). Class I and class II alleles of *daf-2* were defined by their genetic interactions with another mutant *daf-12* in a previous study (Gems et al., 1987). Class II *daf-2* mutants were stronger loss of function alleles than Class I. At least 200 animals were examined in 3 trials. **, $p<0.001$; ns, $p>0.05$ by the Tukey-Kramer comparison test. Error bars, standard deviation.

(B) DAF-16a and DAF-16d/f isoforms regulate dauer formation

Daf-c penetrance of animals of various genotypes at 25ºC. *daf-16* fully suppressed the *Daf-c* phenotype of *daf-2(lf;ts)* mutants. *daf-16(lf;null); daf-2(lf;ts)* animals carrying an integrated transgene that expressed a single DAF-16 isoform, DAF-16a, DAF-16b, or DAF-16d/f, under the endogenous promoter, exhibited difference in *Daf-c* penetrance. Restoring DAF-16a and DAF-16d/f in *daf-16; daf-2* both partially increased its *Daf-c* penetrance. Restoring DAF-16b had no effect. At least 200 animals were examined in 3 trials. **, p<0.001; ns, p>0.05 by the Tukey-Kramer comparison test. Error bars, standard deviation.

Figure S3. Nucleus translation of tissue-specific GFP::DAF-16a transgenes in *daf-2(lf;ts)* mutants

(Left panel) A schematic representation of the canonical insulin signaling pathway. Activation of the insulin/IGF receptor DAF-2 prevents DAF-16 from entering into nuclei. (Right panels) The subcellular localization of the GFP::DAF-16a reporter, expressed specifically in the body wall muscles (by *Pmyo-3*), the nervous system (by *Prgef-1*) and the intestine (by *Pges-1*) in wild-type animals and *daf-2(lf;ts)* mutants. In wild-type animals, all GFP::DAF-16a transgenes exhibit cytoplasmic signals. Reduced insulin signaling activity in *daf-2* mutants leads to constitutive nuclear localization of GFP::DAF-16a in the respective tissues.

Figure S4. DAF-28(gf) does not block INS-4 processing.

(A) A INS-4::GFP reporter, expressed by a panneuronal promoter, was processed in *daf-28(gf;ts)* mutants. (B) A restricted expression of INS-4::GFP in ASI and ASJ (by P*daf-28*) in *daf-28(gf;ts)* mutants also appeared processed.

List of single mutants

Table S1

gene	allele	Comment
daf-2	*e1370*	*lf, ts*
	e1368	*lf, ts*
	m569	*lf, ts*
	e979	*lf, ts*
	e1390	*lf, ts*
kpc-1	*gk8*	*lf, deletion*
egl-3	*ok979*	*lf, deletion*
bli-4	*e937*	*lf*
aex-5	*sa23*	*lf*
daf-16	*mu86*	*lf, deletion*
daf-28	*sa191*	*ts,gf*
	tm2308	*lf*
ins-1	*tm1888*	*lf, deletion*
ins-2	*tm4467*	*lf, deletion*
ins-3	*tm3608*	*lf, deletion*
ins-4	*tm3620*	*lf, deletion*
ins-5	*tm2560*	*lf, deletion*
ins-6	*tm2416*	*lf, deletion*
ins-7	*tm2001*	*lf, deletion*
ins-8	*tm4144*	*lf, deletion*
ins-18	*ok1672*	*lf, deletion*

lf= loss-of-function *gf=gain-of-function* *ts=temperature-sensitive*

List of Plasmids and transgenic lines

Table S2

Plasmid	Description	background	***hpEx***
pJH2292	pan-neural INS-2	*daf-28(gf)*	*2292*
pJH2293	pan-neural INS-3	*daf-28(gf)*	*2372*
pJH2265	pan-neural INS-4	*daf-28(gf)*	*2369*
pJH2356	pan-neural INS-5	*daf-28(gf)*	*2373*
pJH2266	pan-neural INS-6	*daf-28(gf)*	*2370*
pJH2947	pan-neural INS-2::GFP	*wt*	*3337*
		kpc-1	*3338*
		egl-3	*3339*
pJH2916	pan-neural INS-3::GFP	*wt*	*3340*
		kpc-1	*3341*
		egl-3	*3342*
pJH2255	pan-neural INS-4::GFP	*wt*	*2387*
		kpc-1	*3343*
		egl-3	*3344*
		bli-4	
		aex-5	
pJH2256	pan-neural INS-6::GFP	*wt*	*2563*
		kpc-1	*3345*
		egl-3	*3346*
		bli-4	
		aex-5	
pJH1922	pan-neural INS-1::GFP	*wt*	*3102*
		kpc-1	*3348*
		egl-3	*3347*
pJH1498	pan-neural INS-18::GFP	*wt*	*hpIs164*

List of Plasmids and transgenic lines

pJH2741	pan-neural DAF-28::GFP	*wt*	*2876*
		kpc-1	*3349*
		egl-3	*3350*
pJH3029	pan-neural INS-1::GFP A46A47	*wt*	*3351*
pJH3030	pan-neural INS-1::GFP A61A62	*wt*	*3352*
pJH3031	pan-neural INS-1::GFP A46A47A61A62	*wt*	*3353*
pJH2366	pan-neural INS-18::GFP A51A54	*wt*	*2443*
pJH2186	pan-neural INS-18::GFP A64A65	*wt*	*3375*
pJH2848	Pdaf-28-GFP	*wt*	*2974*
pJH3374	Pdaf-28 DAF-28	*hpDf761; daf-28(lf)*	*3505*
pJH3188	Punc-25 DAF-28	*hpDf761; daf-28(lf)*	*3355*
pJH3189	Pacr-2 DAF-28	*hpDf761; daf-28(lf)*	*3356*
pJH2894	INS-4::GFP	*wt*	*2999*
pJH2854	Pdaf-28 INS-4::GFP	*hpDf761; daf-28(lf)*	*3357*
pJH2929	Punc-25 INS-4	*hpDf761; daf-28(lf)*	*3316*
pJH3278	Pacr-2 INS-4	*hpDf761; daf-28(lf)*	*3315*
pJH2854+2929	Pdaf-28 INS-4::GFP+Punc-25 INS-4	*hpDf761; daf-28(lf)*	*3317*
pJH3375	Pdaf-28 INS-6	*hpDf761; daf-28(lf)*	*3506*
pJH2974	Punc-25 INS-6	*hpDf761; daf-28(lf)*	*3359*
pJH3190	Pacr-2 INS-6	*hpDf761; daf-28(lf)*	*3314*
pJH3256	INS-1 SL2 GFP	wt	*3284*
pJH3255	INS-18 SL2 GFP	wt	*3281*

List of Plasmids and transgenic lines

pJH3257	Pges-1 INS-1	*ins-18; ins-1; daf-28(gf)*	*3285*
pJH3258	Pges-1 INS-18	*ins-18; ins-1; daf-28(gf)*	*3360*
pJH1498	pan-neural INS-18::GFP	*ins-18; ins-1; daf-28(gf)*	*3361*
pJH1922	pan-neural INS-1::GFP	*ins-18; ins-1; daf-28(gf)*	*3362*
pJH1464	Pmyo-3 INS-18::RFP	*ins-18; ins-1; daf-28(gf)*	*3510*
pJH2124	pan-neural EGL-3	*egl-3 daf-28(lf)*	*3365*
pJH3058	Pges-1 EGL-3	*egl-3 daf-28(lf)*	*3276*
pJH3376	Pmyo-3 EGL-3	*egl-3 daf-28(lf)*	*3511*
pJH3113	pan-neural KPC-1 mini-gene	*kpc-1; daf-28(lf)*	*3366*
pJH3149	Pges-1 KPC-1 mini-gene	*kpc-1; daf-28(lf)*	*3362*
pJH3252	KPC-1 SL-2 RFP	*wt*	*3368*
pJH1630	Pdpy-30 DAF-2	*daf-2(e1370)*	*2908*
pJH616	Pmyo-3 DAF-2	*daf-2(e1370)*	*2905*
pJH664	pan-neural DAF-2	*daf-2(e1370)*	*2906*
pJH668	Pges-1 DAF-2	*daf-2(e1370)*	*3369*
pJH3043	Pdpy-30 DAF-16a	*daf-16(mu86); daf-2(e1370)*	*3370*
pJH3185	pan-neural GFP::DAF-16a	*daf-16(mu86); daf-2(e1370)*	*3371*
pJH2972	Pmyo-3 GFP::DAF-16a	*daf-16(mu86); daf-2(e1370)*	*3372*
pJH2973	Pges-1 GFP::DAF-16a	*daf-16(mu86); daf-2(e1370)*	*3373*
pJH3377	Pges-1 DAF-16d/f	*daf-16(mu86); daf-2(e1370)*	*3507*
pJH2973+pJH3377	Pges-1 GFP::DAF-16a,d/f	*daf-16(mu86); daf-2(e1370)*	*3508*
pJH2973+pJH3377	Pges-1 GFP::DAF-16a,d/f	*daf-16(mu86); hpDf761; daf-28(lf)*	*3509*

List of Plasmids and transgenic lines

pJH2606	MosDel targeting construct for ins-4 to ins-6	*n/a*	*hpDf761*
